# Development of a Duplex LAMP Assay with Probe-Based Readout for Simultaneous Real-Time Detection of *Schistosoma mansoni* and *Strongyloides* spp. -A Laboratory Approach to Point-Of-Care

**DOI:** 10.3390/ijms24010893

**Published:** 2023-01-03

**Authors:** Beatriz Crego-Vicente, Pedro Fernández-Soto, Juan García-Bernalt Diego, Begoña Febrer-Sendra, Antonio Muro

**Affiliations:** Infectious and Tropical Diseases Research Group (e-INTRO), Biomedical Research Institute of Salamanca Research Centre for Tropical Diseases at the University of Salamanca (IBSAL-CIETUS), Faculty of Pharmacy, University of Salamanca, 37007 Salamanca, Spain

**Keywords:** LAMP, multiplex LAMP, duplex LAMP, DARQ-LAMP, *Schistosoma mansoni*, *Strongyloides* spp., diagnostic, point-of-care

## Abstract

Loop-mediated isothermal amplification (LAMP) is the most popular technology for point-of-care testing applications due its rapid, sensitive and specific detection with simple instrumentation compared to PCR-based methods. Many systems for reading the results of LAMP amplifications exist, including real-time fluorescence detection using fluorophore-labelled probes attached to oligonucleotide sequences complementary to the target nucleic acid. This methodology allows the simultaneous detection of multiple targets (multiplexing) in one LAMP assay. A method for multiplexing LAMP is the amplification by release of quenching (DARQ) technique by using a 5′-quencher modified LAMP primer annealed to 3′-fluorophore-labelled acting as detection oligonucleotide. The main application of multiplex LAMP is the rapid and accurate diagnosis of infectious diseases, allowing differentiation of co-infecting pathogens in a single reaction. Schistosomiasis, caused among other species by *Schistosoma mansoni* and strongyloidiasis, caused by *Strongyloides stercoralis*, are the most common helminth-parasite infections worldwide with overlapping distribution areas and high possibility of coinfections in the human population. It would be of great interest to develop a duplex LAMP to detect both pathogens in the same reaction. In this study, we investigate the use of our two previously developed and well-stablished LAMP assays for *S. mansoni* and *Strongyloides* spp. DNA detection in a new duplex real-time eight-primer system based on a modified DARQ probe method that can be performed in a portable isothermal fluorimeter with minimal laboratory resources. We also applied a strategy to stabilize the duplexed DARQ-LAMP mixtures at room temperature for use as ready-to-use formats facilitating analysis in field settings as point-of-care diagnostics for schistosomiasis and strongyloidiasis.

## 1. Introduction

Nucleic acids are commonly used as important biomarkers for biological studies and medical diagnosis. Polymerase chain reaction (PCR) was the first method for detecting minute quantities of nucleic acids and is still used widely today because it is sensitive, specific and can be quantitative. However, PCR and PCR-based methods have several limitations, mainly due to the susceptibility to inhibitors present in environmental and clinical samples, the need for accurate thermocycling requirements and the difficulty of its applicability in field conditions. These shortcomings prompted the emergence of a number of isothermal nucleic acid amplification assays as a promising alternative to PCR-based methods since the early 1990s onwards. The isothermal amplification methods have gained attention because the advantages compared to PCR-based methods due to their point-of-care (POC) use adaptation by providing simple, fast and low-cost devices to be performed potentially in low-resource settings [1,2]. A chronological development of isothermal amplification methods as well as amplification mechanisms has been reviewed and discussed recently by Glökler et al. [3]. In that timeline, amongst the most frequent and promising isothermal amplification methods applied as potential POC tools are: strand displacement amplification (SDA) [4], helicase dependent amplification (HDA) [5], rolling circle amplification (RCA) [6], recombinase polymerase amplification (RPA) [7], nucleic acid sequence based amplification (NASBA) [8] and loop-mediated isothermal amplification (LAMP) [9].

Since its original report in 2000, LAMP has been the most popular technology among researchers in terms of development and implementation, accounting for roughly 60% of all isothermal amplification assays publications [10]. LAMP allows exponential DNA amplification by stand-displacing polymerase using four to six specific primers to recognize up to six to eight sequences on the DNA template in the same reaction, compared to only two in typical PCR [9]. In combination with reverse transcriptase, LAMP can also be used for RNA amplification (RT-LAMP) [11]. Amplification and detection of nucleic acid (DNA or RNA) by LAMP has important advantages compared to PCR-based methods such as shorter reaction time (30 min vs. 3 h for PCR protocol) [12,13,14,15] and the possibility of using LAMP in POC devices, thus reducing or eliminating the use of electric power, technical equipment and expertise [16,17]. Moreover, there are a large number of possibilities and systems for reading and analyzing the results of LAMP amplifications. On the one hand, readout methods include the typical end-point analysis by agarose gel electrophoresis [9], simple naked eye colorimetric visualization by intercalating dyes [18] or metal indicators [19]. On the other hand, real-time detection is also possible in different devices by turbidity [20], adding color-change of fluorescence metal-sensitive indicators [18], unselective DNA fluorescent intercalating dyes [21] or target specific-fluorogenic probes [10]. Other approaches such as lateral flow assays (LFA) [22] and novel combinations with CRISPR-Cas Systems [23] have also been developed.

At present, real-time fluorescence is a widely used method for the detection of LAMP results, because it is very sensitive, compatible with most standard isothermal amplification devices available in labs and very useful for optimizing and checking the kinetics of amplification reactions. More specifically, target sequence-specific detection using fluorogenic probes ensures outstanding specificity towards the target, thus avoiding the detection of unspecific products accountable for false positives. Besides, this method opens the possibility for the simultaneous detection of multiple targets (multiplexing) in one LAMP assay [10]. The main application of multiplex LAMP (mLAMP) is the rapid and accurate diagnosis of infectious diseases, allowing differentiation not only of multiple species of pathogens, but also of strains or closely related species in a single assay [18,24]. Currently, a number of methods exist for multiplexing LAMP which use fluorophore-labelled probes attached to oligonucleotide sequences complementary to the target nucleic acid, including methylation-specific LAMP (MS-LAMP), assimilating probe-LAMP, fluorescence of loop primer upon self-enriching-LAMP (FLOS-LAMP), detection of amplification by release of quenching (DARQ), quenching of unincorporated amplification signal reporters (QUASR) and molecular beacon LAMP (MB-LAMP). These multiplexed LAMP methods based on fluorogenic probes have been extensively reviewed by Becherer et al. [10]. In general, in such approaches, the type of fluorophore used and the interactions between fluorophores and primers can negatively affect to the fluorescence emission, therefore the fluorophore-labelled probes must be adapted to different target sequences. This is possibly a weakness of the methodology as it requires time-consuming work for primers optimization and probe design to fine-tune the mLAMP assay. Possibly, this is the reason why published studies on multiplexed fluorescence-based detection of LAMP are still scarce and poorly reproduced.

Tanner et al. [25] reported the DARQ technique, in which multiplex detection of LAMP amplification does not need additional primer optimization or probe design, requiring only the use of a 5′-quencher modified LAMP primer (Q-FIP; forward internal primer) annealed to 3′-fluorophore-labelled (Fd) acting as detection oligonucleotide. The strand displacement activity of the *Bst* DNA polymerase displaces the fluorophore-labelled strand from the quencher- labelled primer (or vice versa) during polymerization, allowing a colour real-time multiplexed LAMP assay. The DARQ method can potentially inhibit polymerization, thereby requiring careful titration of the amount of probe used.

As mentioned, the importance of a mLAMP lies in the possibility of specifically detecting multiple strains or genotypes of the same infectious agents as well as different co-infecting pathogens in a single reaction, thus saving time and resources in the diagnosis of infectious diseases. Approximately 30% of human infections may actually be coinfections and this rate could be as high as 80% in some human communities [26,27]. Coinfections with at least two genetically different infectious agents in the same host have been mainly studied in humans, with a special concern for parasite helminths worms [26,28]. Human helminth infections are among the most common infectious diseases, particularly in developing countries. Schistosomiasis, caused among other species by *Schistosoma mansoni* and soil-transmitted helminths, specifically strongyloidiasis caused by *Strongyloides stercoralis*, is the most common helminth infections worldwide [29]. Moreover, the transmission areas of schistosomiasis and strongyloidiasis largely overlap in tropical and subtropical regions in the human population [30,31,32,33]. Therefore, the possibility of coinfections is high, not only to people living in endemic areas, but also in travelers, migrants and refugees [34,35,36,37]. An early diagnosis accompanied by good treatment helps to limit complications. Thus, it would be of great interest to develop a mLAMP to detect both pathogens in the same reaction.

In this study, we investigate the use of our previously well-stablished conventional LAMP assays for *Schistosoma mansoni* DNA detection [38] and for *Strongyloides* spp. DNA detection [39] in a new duplex real-time eight-primer system based on a modified DARQ probe method. The duplex DARQ-LAMP can be performed and readout accomplished in a portable isothermal fluorimeter with minimal laboratory resources. We also applied a strategy to stabilize the duplexed DARQ-LAMP mixtures at room temperature for use as ready-to-use formats facilitating analysis in field settings as POC diagnostics for schistosomiasis and strongyloidiasis.

## 2. Results

### 2.1. Setting up and Operation of DARQ-LAMP

**Figure 1:**
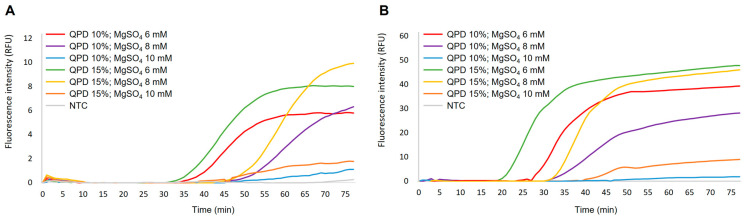
Setting up simplex DARQ-LAMP assays. (**A**) *Schistosoma mansoni* DARQ-LAMP. (**B**) *Strongyloides venezuelensis* DARQ-LAMP. Evaluation of 15% and 10% percentages of quencher probe duplex (QPD) in relation to the total amount of unlabeled FIP and different supplementary MgSO4 concentrations (4, 6 and 8 mM) to obtain final MgSO4 concentrations of 6, 8 and 10 mM are indicated. NTC, non-template control (ultrapure water instead gDNA); RFU, relative fluorescence units.

Figure 1 shows the results of setting up the simplex DARQ-LAMP reaction for amplification of gDNA from *Schistosoma mansoni* (5 ng/µL) and *Strongyloides venezuelensis* (5 ng/µL) using different percentages of quencher probe duplex (QPD) in relation to the total amount of unlabeled FIP and supplementary MgSO_4_ concentrations. Best results for amplification of *S. mansoni* (Figure 1A) and *S. venezuelensis* (Figure 1B) gDNA were obtained when both QPD percentages of 10% (90:10) and 15% (85:15) in relation to the total amount of unlabeled FIP in combination with total MgSO_4_ 6 mM were used at 61 °C. Amplification trials using different QPD/MgSO_4_ combinations at 63 °C, 65 °C and 70 °C showed irregular and non-reproducible amplifications. Overall, the combination of 15% QPD/MgSO_4_ 6 mM produced a better amplification than 10% QPD/MgSO_4_ 6 mM, in terms of time-to-positivity (Tp) (33 min vs. 35 min for *S. mansoni* and 22 min vs. 27 min for *S. venezuelensis*) and in relative fluorescence units (RFU) values in fluorescence intensity measurements (8 RFU vs. 5.8 RFU for *S. mansoni* and 45 RFU vs. 35 RFU for *S. venezuelensis*). In general, for *S. venezuelensis* gDNA amplification, Tp was shorter and fluorescence intensity higher than those for *S. mansoni*. The combination of 15% QPD/MgSO_4_ 6 mM in the presence of *Bst* 2.0 WS at 61 °C was chosen as the most suitable to incorporate into all successive DARQ-LAMP mixtures for *S. mansoni* and *S. venezuelensis* amplification.

### 2.2. Sensitivity Assessment of Simplex DARQ-LAMP Assays

**Figure 2:**
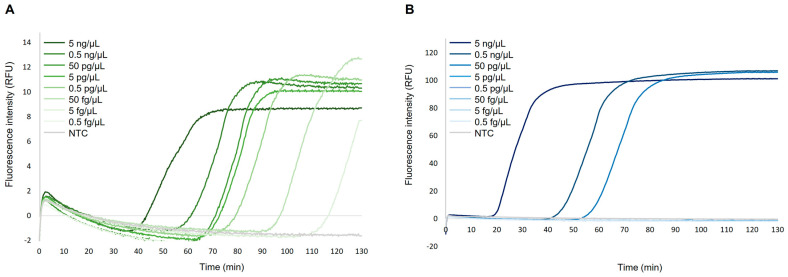
Sensitivity assessment of simplex DARQ-LAMP assays using the Genie III handheld device. (**A**) Analytical sensitivity for *Schistosoma mansoni* DARQ-LAMP reaction. (**B**) Analytical sensitivity for *Strongyloides venezuelensis* DARQ-LAMP reaction. The 10-fold serial dilutions (5 ng/µL to 5 fg/µL) of gDNA from parasites are represented by different shades of green (for *Sm*) and blue (for *Sv*). NTC, non-template control (ultrapure water instead gDNA) is represented by grey lines in both cases. RFU, relative fluorescence units.

Figure 2 shows the different amplification time and fluorescence intensity in sensitivity tests using 10-fold serial dilutions of gDNA from *S. mansoni* and *Strongyloides venezuelensis*. The limit of detection in *S. mansoni* gDNA DARQ-LAMP amplification was 5 fg/µL at 116 min (Figure 2A). The limit of detection in *S. venezuelensis* gDNA DARQ-LAMP amplification was 50 pg/µL at 52 min (Figure 2B). In general, Tp and RFU values were clearly shorter and higher, respectively, for amplification of *S. venezuelensis* gDNA than for *S. mansoni*. As expected, higher concentrations of parasite gDNA were amplified more quickly, but in any case always with longer amplification times for *S. mansoni* than for *S. venezuelensis*.

### 2.3. Operation of Duplex DARQ-LAMP Assay

**Figure 3:**
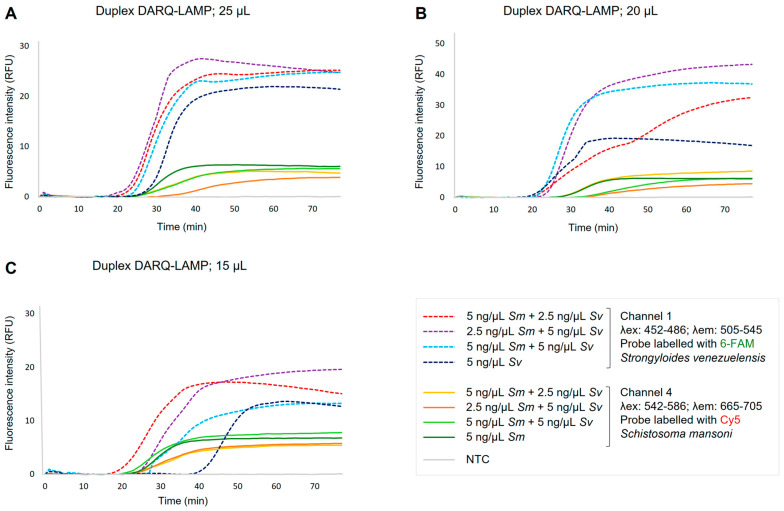
Duplex DARQ-LAMP for simultaneous amplification of gDNA from *Schistosoma mansoni* and *Strongyloides venezuelensis* using different combinations of gDNA concentrations as template in different volumes of master mixes. (**A**) Duplex DARQ-LAMP in 25 µL. (**B**) Duplex DARQ-LAMP in 20 µL. (**C**) Duplex DARQ-LAMP in 15 µL. Combinations of gDNA concentrations of the two parasites used as templates are indicated on the right panel. NTC, non-template control (ultrapure water instead gDNA). The reactions were carried out in a PCR max Eco 48 Real Time PCR System. Dotted lines indicate fluorescence reading in channel 1 for *Strongyloides venezuelensis* amplification (probe labelled with 6-FAM) and continuous lines indicate fluorescence reading in channel 4 for *Schistosoma mansoni* amplification (probe labelled with Cy5).

Duplex DARQ-LAMP worked well, allowing amplification of both parasites’ gDNA simultaneously when using different combinations of gDNA concentrations in different volumes of master mixes (Figure 3). In the duplex assays carried out with a reaction volume of 25 µL (Figure 3A) and 20 µL (Figure 3B), the amplification profile was very similar for both parasites to that obtained in simplex DARQ-LAMP reactions, showing for *S. venezuelensis* values of Tp and fluorescence intensity shorter and higher, respectively, than for *S. mansoni*. A slight decrease in fluorescence intensity for both parasites was observed when the minimum volume of 15 µL (Figure 3C) was used for the reaction mixtures; in any case, amplification occurred and, moreover, distinguished well between the two parasites. Notwithstanding this, the Tp for *S. venezuelensis*-specific amplification increased in at least 20 min.

### 2.4. Sensitivity Assessment of Duplex DARQ-LAMP Assay

**Figure 4:**
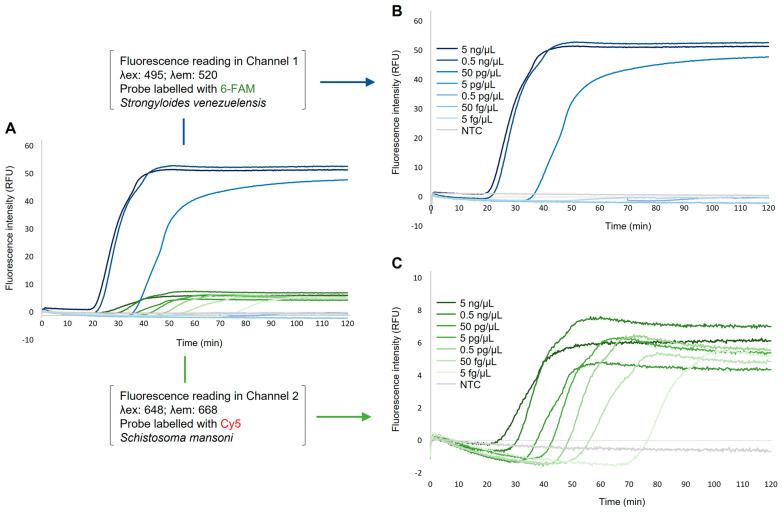
Sensitivity assessment of duplex DARQ-LAMP assay using the Genie III handheld device. (**A**) Analytical sensitivity for simultaneous detection of *Schistosoma mansoni* gDNA and *Strongyloides venezuelensis* gDNA using 10-fold serial dilutions. (**B**) Analytical sensitivity for *S. venezuelensis* in duplex DARQ-LAMP reaction. Only the excitation (λex = 495) and emission (λem = 520) channel 1 for fluorescence measurement of 6-FAM is represented. (**C**) Analytical sensitivity for *S. mansoni* in duplex DARQ-LAMP reaction. Only the excitation (λex = 648) and emission (λem = 668) channel 2 for fluorescence measurement of Cy5 is represented. The 10-fold serial dilutions (5 ng/µL to 5 fg/µL) of gDNA from parasites are represented by different shades of blue (for *S. venezuelensis*) and green (for *S. mansoni*). NTC, non-template control (ultrapure water instead gDNA) is represented by grey lines in both cases. RFU, relative fluorescence units.

Once the proper operation of duplex DARQ-LAMP assay was verified, the sensitivity was attempted in the Genie III handheld device using 10-fold serial dilutions prepared by mixing those 10-fold serial dilutions used in the simplex DARQ-LAMP sensitivity assays (Figure 4). When running the duplex DARQ-LAMP, the limit of detection for *S. mansoni* gDNA was 5 fg/µL (Tp~77 min) and for *S. venezuelensis* gDNA resulted in 50 fg/µL (Tp~36 min). For a better visualization of the 10-fold serial dilution amplifications in dual excitation and emission channels in Genie III handheld device, the measured fluorescence of QPDs is represented in two separate graphs: one for fluorescence reading in channel 1 for *Strongyloides venezuelensis* (Figure 4B) and another for fluorescence reading in channel 2 for *Schistosoma mansoni* (Figure 4C). As in the evaluation of sensitivity in simplex DARQ-LAMP reactions, when performing the duplex DARQ-LAMP assay, the Tp and RFU values were clearly shorter and higher, respectively, for *S. venezuelensis* amplification than for *S. mansoni.* It should be noted that the detection limit for each parasite in the duplex DARQ-LAMP assay was identical to that obtained individually in the simplex DARQ-LAMP assays.

### 2.5. Stability and Functionality over Time of Duplex Dry-DARQ-LAMP

**Figure 5:**
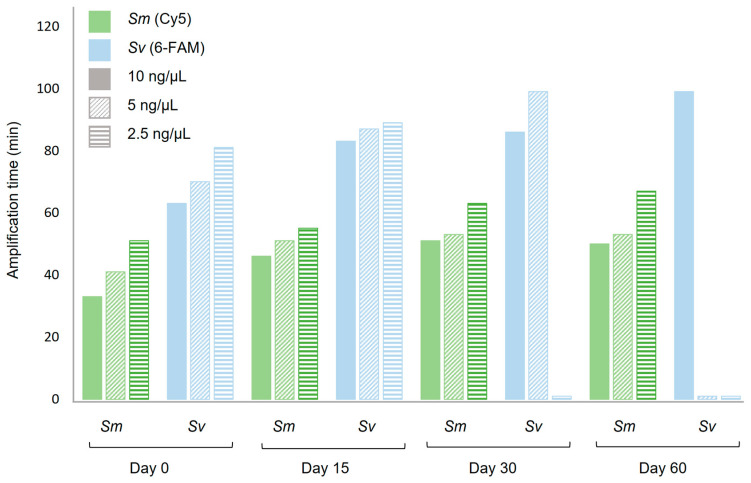
Amplification time of duplex Dry-DARQ-LAMP assays as a function of storage time at ambient temperature. Amplification times of different concentrations of gDNA (10 ng/µL, 5 ng/µL and 2.5 ng/µL) from both *Schistosoma mansoni* (*Sm*) and *Strongyloides venezuelensis* (*Sv*) in duplex DARQ-LAMP assays performed with dry mixtures containing sets of primers and fluorescence labelled-probes tested at 0, 15, 30 and 60-days post-desiccation are represented. Al reactions were performed in a Genie III portable instrument.

The results obtained after the reconstitution of duplex Dry-DARQ-LAMP master mixes for simultaneous amplification of *S. mansoni* and *S. venezuelensis* are shown in Figure 5. In general, after storage for 0, 15, 30 and, 60 days post-desiccation at RT, the reconstitution of dry master mixes was found to be functional for duplex amplifications, although a delay in amplification time during the reaction for dried components was observed in comparison to fresh duplex DARQ-LAMP mixtures. As a general rule, irrespective of the concentration of amplified gDNA from the two parasites, there was an increase in Tp values as storage time increased. Just after drying of the mixtures (on day 0) an increase in Tp was observed for the amplification of *S. mansoni*. This increase was even greater for the amplification of *S. venezuelensis* despite amplifying with much shorter Tp than *S. mansoni* in fresh DARQ-LAMP duplex. The amplification of *S. mansoni* using the duplex dry-DARQ-LAMP format was reasonably stable during 60 days of storage and did not undergo a large increase in Tp over time. Furthermore, amplification was obtained up to 60 days post-desiccation with the lowest gDNA concentration tested (2.5 ng/mL). For *S. venezuelensis*, the lowest DNA concentrations did not show amplification after 30 days of storage (2.5 ng/µL) and 60 days of storage (5 ng/µL and 2.5 ng/µL).

## 3. Discussion

In this study, we have developed a duplex LAMP assay for the simultaneous specific detection of *Schistosoma mansoni* and Strongyloides spp. infection, based on a modified DARQ probe method [25]. The duplex DARQ-LAMP was based on two well-stablished conventional LAMP assays previously described by our group and successfully tested on samples from an experimental model of schistosomiasis [38] and strongyloidiasis [39] as well as on clinical samples, including application in field conditions [40,41,42].

First, in standardization of simplex DARQ-LAMP reactions, the best amplification results in terms of Tp and RFU values for both *S. mansoni* and *Strongyloides* spp. were obtained with a combination of 15% QPD (85:15 unlabelled primer to QPD) and MgSO_4_ 6 mM in the presence of *Bst* 2.0 WS at 61 °C. In the DARQ-LAMP method, the QPD has been previously reported to show amplification inhibition because of the hybridization of the complementary quencher to the incorporating primer prior to the start of the reaction to be displaced during the course of amplification, therefore generating a signal [25,43,44]. As pointed out by Tanner et al. [25,43] and later by Nanayakkara et al. [45], DARQ-LAMP reactions using a 1:1 ratio of unlabeled inner primer (FIP) and QPD result in an inhibition of the amplifications. In order to avoid this inhibition, we conducted a titration of the QPD (15% and 10%) into LAMP reactions with both the *S. mansoni* and *Strongyloides* spp. primer sets to determine optimal conditions for the assays. In addition, as also noted by Tanner et al. (2014) [43], some amount of unlabeled inner primer (FIP) must be present in the reaction to prevent full inhibition of amplification. Thus, regardless of the QPD and FIP ratio used (15% or 10%), a concentration of 1.6 µM was always maintained at in all reactions tested.

With 15% QPD, but also with 10% QPD, in combination with higher concentrations than MgSO_4_ 6 mM, we observed either an increase in amplification times (when using MgSO_4_ 8 mM) or a near-total inhibition of amplification (when using MgSO_4_ 10 mM). With 10% QPD in combination with MgSO_4_ 6 mM, an optimal amplification was also obtained, but we observed a small decline in the amplification curve (just over two RFU units: 5.8 RFU vs. 8 RFU for *S. mansoni* and up to ten RFU units: 45 RFU vs. 35 RFU for *Strongyloides* spp.) and a slight increase in Tp (2 min: 33 min vs. 35 min for *S. mansoni* and 5 min: 22 min vs. 27 min for *Strongyloides* spp.) in comparison to the best values obtained with 15% QPD and MgSO_4_ 6 mM. Curiously, a different effect of the titration of QPD was observed by Nanayakkara & White [45] in amplification of three genes present in the methicillin-resistant *Staphylococcus aureus* (MSRA) genome using DARQ-LAMP method. In that work, using 15% QPD, an increase in amplification time in comparison to 10% QPD was observed. In addition, the authors concluded that the DARQ-LAMP is best suited for assays that require a high Mg^2+^ concentration. By contrast, in our work, our DARQ-LAMP assays performed better with lower MgSO_4_ concentrations (6 mM) than those used in the conventional original LAMP assays (8 mM) described by us for the detection of the two parasites [38,39]. Thus, for a proper DARQ-LAMP operation, it is very important not only to determine the optimal amount of QPD but also the MgSO_4_ concentration for each type of LAMP test to be used.

In sensitivity tests for simplex DARQ-LAMP assays, the *S. mansoni* DARQ-LAMP showed a limit of detection of 5 fg/µL, whereas the *Strongyloides* spp. DARQ-LAMP showed a limit of detection of 50 pg/µL. The sensitivity of amplification based on DARQ probe method was lower than the conventional colorimetric LAMP assays using SYBR green I as intercalator dye originally described by us for *S. mansoni* (1 fg) [38] or *Strongyloides* spp. (10 pg) [39]. This decrease in sensitivity could be due to the inhibitory effect described for DARQ method, as previously observed in the amplification of several other targets [45]. To reduce inhibition in the DARQ technique, other versions of this approach have been used, moving the quencher and fluorophore to a loop primer [46,47] or incorporated into the amplification products and detected after the reaction, giving rise to the QUASR method [44].

When the two set of DARQ-LAMP primers were combined in the same reaction for the duplex DARQ-LAMP assay, we obtained a very satisfactory performance, with no detectable cross-reactivity or non-specific amplifications of the negative controls and with amplification times and fluorescence signals virtually identical to those obtained in the simplex DARQ-LAMP assays. In addition, it should be noted that specificity for the two conventional LAMP assays was previously proven in the original studies in which they were first described [38,39]. Moreover, the duplex DARQ-LAMP was shown to be operative in different reaction volumes as 25 µL, 20 µL and even 15 µL (in the latter case with a slight decrease in fluorescence intensity for *Strongyloides* spp., but well distinguishable from *S. mansoni*). The advantage of operating at such low volumes is that the amount of reagents is reduced and, therefore, the cost per reaction is significantly lower. Furthermore, with regard to the sensitivity of the duplex DARQ-LAMP assays, despite the slight decrease in sensitivity obtained in each of the simplex DARQ-LAMP assays compared to the conventional LAMP assays, these sensitivity values were maintained when combining the two sets of primers and labelled probes in the same reaction in duplex format. Accordingly, the duplex assay had the same amplification yield as the separate singleplex assays.

Once the proper operation was verified, in order to develop a duplex DARQ-LAMP method as simply as possible to carry out detection in any condition for *S. mansoni* and *Strongyloides* spp., we tried to preserve all necessary components in a non-reactive state using standard tubes containing dry master mixes (duplex dry-DARQ-LAMP). To do so, we applied a simple and optimized one-step dry-up desiccation procedure previously reported by our group for drying LAMP reagents adapted for conventional and real-time amplification assays [14,48]. The one-step dry-up protocol was applied for DARQ-LAMP mixtures containing the sets of primers for *S. mansoni* and *Strongyloides* spp., resulting in functional amplifications of both gDNAs after storage at ambient temperature (25 °C) for up to 15, 30 and 60-days post-desiccation. Nevertheless, an increase in amplification times and a reduction in fluorescence signals were observed compared to the fresh liquid DARQ-LAMP mixtures. Although the reactions were functional, it should be noted that the longer dry components are stored at ambient temperature, the longer the reaction incubation time to achieve amplification. This result has already been noted by our group when working with dry-LAMP mixtures [14,48].

Interestingly, in the dry-DARQ-LAMP duplexes tested, this effect was much more remarkable in *Strongyloides* spp. amplification than for *S. mansoni*. For the same concentrations of gDNA from the two parasites, *Strongyloides* spp. amplification required longer amplification time with a lower signal intensity than *S. mansoni* amplification. In addition, amplification was no longer achieved at the lowest *Strongyloides* spp. gDNA concentrations (2.5 ng/µL and 5 ng/µL) at 30 and 60-days post-desiccation, respectively. In any case, very reasonable amplification time of 99 min was observed (for 10 ng/µL) after storage in dry format for 60 days. This is a very surprising result considering that in duplexed DARQ-LAMP using fresh mixtures, for *Strongyloides* spp. the Tps were shorter and the RFUs higher than those obtained for *S. mansoni*. The absence of information on this or similar event in the already published data does not allow us to compare our results. At the moment, we can only speculate on the possibility that some features related to quenched primer or fluorophore-labeled probe used for *Strongyloides* spp. amplification may be affected more by the drying process of the mixtures than those used for *S. mansoni*. This issue needs to be studied further. Despite this, maintaining functionality for at least 60 days without the need for a cold chain would allow us to prepare and distribute within several weeks a set of dry-DARQ-LAMP mixtures in a ready-to-use format for use in portable devices with real-time fluorescence readout useful in field diagnostic tests for schistosomiasis and strongyloidiasis in resource-limited areas. Having dry mixes in a single tube greatly facilitates work in the field and avoids possible contamination. To the best of our knowledge, this is the first time that pre-mixed dried LAMP assays using the DARQ-LAMP technology are developed for simultaneous detection of two different human pathogens. This methodology could be used to develop other duplex DARQ-LAMP formats useful in the detection of other infectious agents.

## 4. Materials and Methods

### 4.1. Parasites DNA Standard Samples

For amplification trials, genomic DNA (gDNA) from *Schistosoma mansoni* (*Sm*) and *Strongyloides venezuelensis* (*Sv*) was extracted from frozen adult worms and infective third-stage larvae (iL3), respectively, using NucleoSpin Tissue Kit (Macherey-Nagel, GmbH & Co., Düren, Germany) following the manufacturers’ instructions. These parasites are routinely maintained by serial passages in laboratory mice (for *Sm*) and rats (for *Sv*) experimentally infected in the Laboratory of Parasitic and Molecular Immunology, CIETUS, University of Salamanca, Salamanca, Spain. The gDNA concentration of the parasites was measured three times by spectrophotometry using a Nanodrop ND-100 spectrophotometer (Nanodrop Technologies, Wilmington, DE, USA) to obtain an average concentration and subsequently 10-fold diluted with ultrapure water to different concentrations for all experiments, as further indicated for each situation. The gDNA samples used for simplex-LAMP reactions only contained gDNA from one species, either *Sm* or *Sv*. For duplex-LAMP reactions, a combination of both parasites gDNA (*Sm* + *Sv*) was used. All gDNA samples were stored at −20 °C until use.

### 4.2. DARQ LAMP Primer Design

LAMP primer sets used were those previously described elsewhere by our group for detection of species-specific *Schistosoma mansoni* based on a 620 base pair (bp) sequence corresponding to a mitochondrial minisatellite DNA region (GenBank Accession No. L27240) [38] and for *Strongyloides* spp., based on a 329 bp sequence corresponding to a linear genomic DNA partial sequence in the 18S rRNA gene from *Strongyloides venezuelensis* (GenBank Accession No. AJ417026.1), which has 94–99% similarity with other sequences reported for *Strongyloides* spp., including the species causing human strongyloidiasis, *S. stercoralis* and *S. fuelleborni* [39].

**Figure 6:**
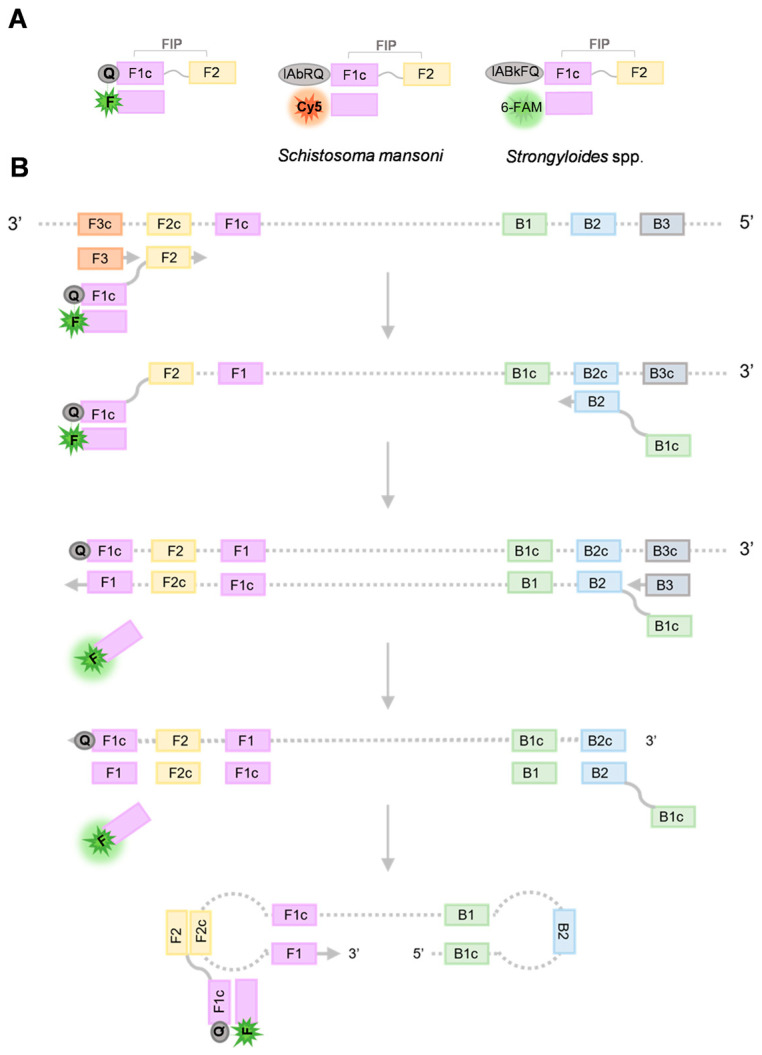
Schematic illustration of customized primers and the principle of the DARQ-LAMP technique. (**A**) Schematic illustration of a Quencher Probe Duplex (QPD), with a 5′-quencher FIP (Q-F1c + F2 sequence) annealed to a sequence complementary to F1c labeled at the 3′ end with fluorophore (Fd). In this study, from each set of primers used, both FIP primers (comprising F1c + F2 sequences) were labelled at 5′ end by adding a dark quencher (Q-FIP): Iowa Black RQ (IAbRQ) for *Schistosoma. mansoni*-FIP primer and Iowa Black FQ (IAbFQ) for *Strongyloides* spp.-FIP primer. F1c sequences complementary probes were designed and labelled with a fluorophore at 3′ end: Cyanine 5 (Cy5) and 6-Carboxyfluorescein (6-FAM) for amplification of *S. mansoni* and *Strongyloides* spp., respectively. (**B**) Schematic diagram of DARQ-LAMP operation, with LAMP inner primers FIP (F1c + F2) and BIP (B1c + B2) and outer primers F3 and B3 and the QPD (Q-FIP + Fd). (1) LAMP is initiated at the F2c sequence of the DNA target, with the Fd probe turned off by hybridization to Q-FIP. This new strand is displaced by upstream synthesis from the external primer F3. (2) The BIP primer (B1c + B2) is anchored to the B2c sequence on the newly synthesized strand. (3) Synthesis from the primer annealed to the B2c sequence displaces the Fd probe. This releases the quenching generating a fluorescent signal. The newly synthesized strand is displaced by the extension from the external primer B3. (4) The resulting structure undergoes exponential amplification in the LAMP reaction. Successive initiations at primer FIP result in additional release of Fd, resulting in exponential detection of the signal.

From each set of primers, both FIP primers (comprising F1c + F2 sequences) were labelled at 5′ end by adding a dark quencher (Q-FIP): Iowa Black RQ (IAbRQ) for *S. mansoni*-FIP primer and Iowa Black FQ (IAbFQ) for *Strongyloides*-FIP primer. F1c sequences complementary probes were designed and labelled with a fluorophore at 3′ end: Cyanine 5 (Cy5) and 6-Carboxyfluorescein (6-FAM) for amplification of *S. mansoni* and *Strongyloides* spp., respectively. Q-FIP primers and their complementary dye labelled probes were synthetized by Integrated DNA Technologies (Coralville, IA, USA) and F3 and B3 primers were synthetized by Eurofins Genomics (GmbH, Ebersberg, Germany). All DNA oligonucleotides (listed in Table 1) were of HPLC grade. The lyophilized primers were resuspended in ultrapure water to a final concentration of 100 µM and stored at −20 °C until use. A schematical illustration of customized primers and the principle of the DARQ-LAMP technique are showed in Figure 6.

### 4.3. DARQ-LAMP Reactions

Set up of DARQ-LAMP reactions were based on original protocols previously described by Tanner et al. [25] and Nanayakkara et al. [45].

First, quenched FIP primer and fluorophore-labelled probe were hybridized (quencher probe duplex, QPD) by incubating the sequences at 50 µM at 95 °C for 2 min at a heat block and slow cooled to room temperature (RT). This stock of QPD was stored at −20 °C until use in the different LAMP assays.

Since each LAMP assay must be optimized to use each modified primer set (by incorporating the additional FIP* primers and the labelled probes into the master mix), different reaction conditions were tested for fine-tuning. On one hand, simplex DARQ-LAMP reactions (i.e., including gDNA from only one parasite, either *Schistosoma mansoni* or *Strongyloides venezuelensis*) were carried out in the presence of *Bst* 2.0 WarmStart DNA Polymerase (*Bst* 2.0 WS) (NEW ENGLAND BIOLABS Ltd., Ipswich, UK) in a volume of 25 µL containing 1.4 mM of each dNTP (BIORON GmBH, Römerberg, Germany), 1× Amplification Buffer (20 mM Tris-HCl (pH 8.8), 50 mM KCl, 10 mM (NH_4_)_2_SO_4_, 2 mM MgSO_4_, 0.1% Tween20), 0.2 μM outer F3/B3 primers, 1.6 µM inner primer BIP and 1.6 µM of a mix including non-labelled inner primer FIP and QPD (FIP* and labelled probe). Reactions were tested with supplementary MgSO_4_ (ranging 6, 8, or 10 mM). Different temperatures (61 °C, 65 °C and 70 °C) and reaction times (60 min, 90 min) were also tested in two different devices with real-time fluorescence reading, a Genie III portable instrument (OPTIGENE Ltd., Horsham, UK) and a PCRmax Eco 48 Real Time qPCR System. The excitation and emission channels for these two devices for real-time fluorescence reading and corresponding fluorophores and quenchers used in DARQ-LAMP reactions are showed in Table 2. To test proper operation of DARQ-LAMP reactions, positive amplification controls (consisting in 5 ng/µL of gDNA template from parasites) and negative controls (consisting in ultrapure water instead gDNA) were used in each trial.

Once simplex DARQ-LAMP reactions were standardized for duplex DARQ-LAMP reactions (i.e., for simultaneous amplification of gDNA from both parasites, *Schistosoma mansoni* and *Strongyloides venezuelensis*), the proportion of primer sets used was 50:50 and the final concentration of the two sets of primers was maintained at 3.6 µM. Thus, to assess first the proper operation of duplex DARQ-LAMP, different combinations of gDNA concentrations in different volumes of master mixes were tested to evaluate the simultaneous amplification of both parasites. We tested combinations of: *Sm* 5 ng/µL + *Sv* 2.5 ng/µL; *Sm* 2.5 ng/µL + *Sv* 5 ng/µL; *Sm* 5 ng/µL + *Sv* 5 ng/µL, and also individual *Sm* 5 ng/µL and *Sv* 5 ng/µL, in different volumes of 25 µL, 20 µL and 15 µL.

In addition, we performed QPD titration in LAMP reactions with the *Schistosoma mansoni* and *Strongyloides* spp. primer sets to determine the optimal assay conditions. For this, from the stock of QPD at 50 µM, a QPD dilution at 5 µM was prepared and percentages of 10% (90:10) and 15% (85:15) in relation to the total amount of unlabeled inner primer (FIP) were used in DARQ-LAMP reactions. Regardless of the ratio of QPD and FIP used, a concentration of 1.6 µM was always maintained in all reactions tested.

### 4.4. Sensitivity of Simplex and Duplex DARQ-LAMP Reactions

The lower detection limit of simplex DARQ-LAMP assays for *S. mansoni* and *S. venezuelensis* gDNA detection was stablished by using 10-fold serial dilutions of gDNA from each parasite, ranging from 5 ng/µL to 0.5 fg/µL.

Sensitivity of duplexed DARQ-LAMP for *Sm* and *Sv* gDNA detection was stablished using 10-fold serial dilutions prepared by mixing those 10-fold serial dilutions used to stablish the sensitivity of simplex tests, thus ranging from mixtures containing 5 ng/µL of *Sm* gDNA and 5 ng/µL of *Sv* gDNA to 5 fg/µL of *Sm* gDNA and 5 fg/µL of *Sv* gDNA.

The tests for the establishment of sensitivity of DARQ-LAMP reactions were carried out in the Genie III handheld device scheduled at 61 °C for 120–130 min.

### 4.5. Stabilization of DARQ-LAMP Reaction Mixes for Room-Temperature Storage: Dry-DARQ-LAMP

For potential ready-to-use DARQ-LAMP multiplex tests, we carried out the stabilization of the master mixes for long-term RT storage following the methodology previously described in our group by García-Bernalt Diego et al. [14]. The stabilization is based in a one-step dry-up vacuum process without centrifugation (so called, desiccation) in a Concentrator Plus (EPPENDORF, Hamburg, Germany) at RT for 30 min. Briefly, DARQ-LAMP master mixes were dried in open 8-tube Genie Strips (OPTIGENE, Horsham, UK) separately in two partial mixes: one containing Isothermal Buffer 10×, MgSO_4_ in the tube cap in the presence of trehalose 2M, the other containing both sets of primers for *S. mansoni* and *Strongyloides* spp., non-labelled inner primers FIP, QPD, dNTPs and *Bst* 2.0 WS polymerase in the bottom of the tube in the presence of trehalose 2M. After the desiccation process, two small, stable and well-adhered pellets in both cap and bottom of the tubes were obtained and, subsequently, the tubes were kept at RT until use. Afterwards, rehydration was carried out with ultrapure water (for negative controls) or ultrapure water containing both *S. mansoni* and *S. venezuelensis* gDNA (for positive controls) and DARQ-LAMP reactions performed with Genie III portable instrument at 61 °C for up to 120 min at 0, 7, 15, 30 and 60-days post-desiccation.

## 5. Conclusions

In summary, a duplex LAMP assay for simultaneous detection of two high prevalent human pathogens, *Schistosoma mansoni* and *Strongyloides* spp., was developed. The differentiation of the multiple amplified targets, based on DARQ technology, was optimized for easily readout in a handheld isothermal fluorimeter. In addition, our one-step dry-up protocol for the stabilization of multiplexed master mixes produces ready-to-use functional formats that can be easily prepared, distributed and applied in resource-limited field settings by untrained personnel, therefore increasing its potential as point-of-care diagnostic method. Further studies are needed to prove its efficiency in the analysis of clinical samples.

## Figures and Tables

**Table 1:** LAMP primer and probe sequences.

Species	Target	Primers	Sequence (5 → 3)	Reference
*Sm*	MIT-Sm	F3	TTATCGTCTATAGTACGGTAGG	Fernández-Soto et al. [38]
		B3	ATACTTTAACCCCCACCAA	
		BIP	AGAAGTGTTTAACTTGATGAAGGGGAAACAAAACCGAAACCACTA	
		FIP	GCCAAGTAGAGACTACAAACATCTTTGGGTAAGGTAGAAAATGTTGT	
		FIP*	**IAbRQ-**GCCAAGTAGAGACTACAAACATCTTTGGGTAAGGTAGAAAATGTTGT	This study
		Probe	AAGATGTTTGTAGTCTCTACTTGGC—**Cy5**	
*Str* spp.	*18S* *rDNA*	F3	ACACGCTTTTTATACCACATT	Fernández-Soto et al. [39]
		B3	GTGGAGCCGTTTATCAGG	
		BIP	ATCAACTTTCGATGGTAGGGTATTGCCTATCCGGAGTCGAACC	
		FIP	ACCAGATACACATACGGTATGTTTTGGATTTGATGAAACCATTTTTTCG	
		FIP*	**IAbFQ-**ACCAGATACACATACGGTATGTTTTGGATTTGATGAAACCATTTTTTCG	This study
		Probe	AAAACATACCGTATGTGTATCTGGT—**6-FAM**	

*Sm*: *Schistosoma mansoni*; MIT-Sm: mitochondrial *S. mansoni* minisatelite DNA region (GenBank Accesion No. L27240). *Str*: *Strongyloides* spp.; *18S rDNA*: linear genomic DNA partial sequence in *18S rDNA* from *Strongyloides venezuelensis* (GenBank Accession No. AJ417026.1). F3, B3: forward and backward external primers; BIP: backward inner primer (comprising B1c and B2 sequences); FIP, forward inner primer (comprising F1c and F2 sequences); FIP*, forward inner primer (comprising F1c and F2 sequences) labeled at 5′ end with an Iowa Black RQ (for *Sm*) or Iowa Black FQ (for *Str*) quencher. Probe: the probe sequences have the designated fluorophore labelled at the 3′ end (Cy5 for *Sm*; 6-FAM for *Str*).

**Table 2:** Excitation and emission channels in Genie III portable instrument and PCRmax Eco 48 Real Time qPCR System used for real-time amplifications and corresponding fluorophores and quenchers used in DARQ-LAMP assays.

Device	Channel	λex	λem	Fluorophore	Quencher
Genie^®^ III	1	470	510–560	6-FAM (λex 495–λem 520)	IAbFQ (420–620)
	2	590	>620	Cy5 (λex 648–λem 668)	IAbRQ (500–800)
PCR max Eco48	1	452–486	505–545	6-FAM (λex 495–λem 520)	IAbFQ (420–620)
	2	452–486	604–644		
	3	542–586	562–596		
	4	542–586	665–105	Cy5 (λex 648–λem 668)	IAbRQ (500–800)

Genie III instrument and PCR max Eco 48 Real Time qPCR System include dual channel and four channel fluorescence measurement, respectively. For DARQ-LAMP assays in PCRmax Eco 48 Real Time qPCR System only channels 1 and 4 were used. λex, fluorescence excitation wavelength; λem, fluorescence emission wavelength. Fluorophore: 6-FAM, 6-Carboxyfluorescein; Cy5, Cyanine 5. Quencher: IAbFQ, Iowa Black FQ for *Strongyloides*-FIP primer; IAbRQ, Iowa Black RQ for *S. mansoni*-FIP primer. λex, λem and quenching range are expressed in nanometers (nm).

## Data Availability

Not applicable.
